# Coping style, social support and psychological distress in the general Chinese population in the early stages of the COVID-19 epidemic

**DOI:** 10.1186/s12888-020-02826-3

**Published:** 2020-08-27

**Authors:** Hua Yu, Mingli Li, Zhixiong Li, Weiyi Xiang, Yiwen Yuan, Yaya Liu, Zhe Li, Zhenzhen Xiong

**Affiliations:** 1 Mental Health Center and National Clinical Research Center for Geriatrics, West China Hospital, Sichuan University, No. 28 Dian Xin Nan Road, Chengdu, 610041 Sichuan China; 2 The Third Department of Clinical Psychology, Karamay Municipal People’s Hospital, Karamay, 830054 Xinjiang China; 3 The West China College of Medicine, Sichuan University, Chengdu, 610041 Sichuan China; 4 School of Nursing, Chengdu Medical College, Chengdu, 610083 Sichuan China

**Keywords:** COVID-19, Psychological distress, Coping style, Social support, Cross-sectional

## Abstract

**Background:**

The purpose of this study was to investigate the psychological status of the general population in mainland China during the outbreak of coronavirus disease 2019 (COVID-19), and to explore the factors influencing psychological distress, in order to provide the basis for further psychological intervention programs.

**Methods:**

We administered three questionnaires on-line to a convenience sample of the general population from different regions of mainland China from February 1 to February 4, 2020. We used the Mandarin versions of the six-item Kessler psychological distress scale (K6), the Simplified Coping Style Questionnaire (SCSQ), and the Social Support Rating Scale (SSRS). We also collected demographic data and other information related to the COVID-19 outbreak. Multivariate binary logistic regression analysis was used to identify factors influencing psychological distress.

**Results:**

Of 1607 respondents, 1588 returned valid questionnaires and were included in the analysis. Nearly one quarter (22.8%) had high levels of psychological distress (K6 score ≥ 13). Individuals with higher psychological distress were more likely to be unmarried, spend more than 6 h per day searching for information about COVID-19, more frequently adopt a passive coping style, and report less social support than those with lower psychological distress.

**Conclusions:**

The COVID-19 outbreak in China has a great impact on the mental health status of the general population. Active coping strategies and increased social support are significantly correlated with decreased psychological distress, and may serve as the basis for psychological interventions.

## Background

An outbreak of infections of severe acute respiratory syndrome coronavirus 2 (SARS-CoV-2), initially called novel coronavirus (2019-nCoV), began on December 8, 2019, when several cases of pneumonia of unknown etiology were reported in Wuhan in Hubei Province of China [1]. In the early stages of this pneumonia, severe acute respiratory infection symptoms can occur, with some patients rapidly developing acute respiratory distress syndrome, acute respiratory failure, and other serious complications [2]. As of March 11, 2020, the total number of patients in China with confirmed coronavirus disease 2019 (COVID-19) was 80,955, of which 67,773 were in Hubei province, and the total number of COVID-19-associated deaths was 3162 [3] . At the end of January 2020, the World Health Organization declared the COVID-19 outbreak in China as a public health emergency of international concern.

Infectious diseases cause significant psychological distress, both in the general public and in health professionals [4]. The emergence of COVID-19 has parallels with the pandemic of human immunodeficiency virus infection and acquired immune deficiency syndrome (HIV/AIDS), the severe acute respiratory syndrome (SARS) outbreak and the threat of an avian influenza pandemic, all of which caused substantial concern among health authorities, the media, and the general public [5]. As a life-threatening disease, we can consider COVID-19 outbreak as a specific stress. Psychosocial responses towards infectious disease outbreaks are variable and can range in intensity, including feelings of anxiety, a sense of shame, failure or weakness of the individual and society; an underestimation of likelihood of survival; an overestimation of likelihood of infection [6]; an urge to take flight from the outbreak; excessive, inappropriate adoption of precautionary measures; and increased demand for healthcare services during a critical shortage [7]. Very few epidemiological data are available about mental health problems and psychiatric morbidity among those suspected or diagnosed with COVID-19, the health professionals treating them and the general population [8]. Therefore, the best strategies to respond to mental health challenges during the outbreak remain unknown.

Previous research has indicated that coping styles and social support are moderating variables in the relationship between stress and distress [9], and that the relationship among stress, coping strategy and support system is complicated [10, 11]. A framework has been constructed in the support/coping field, in which social support and coping strategy have main effects on stress without a significant interaction between them [12]. Coping strategies refer to the specific efforts, both behavioral and psychological, that people employ to master, tolerate, reduce, or minimize stressful events [13]. During exposure to stressors, different individuals, or the same individual under different conditions, can employ active or passive coping strategies [14]. Active coping strategies include (1) considering ways to overcome stress and make plans for subsequent efforts, (2) accepting the existence of stressful events, and (3) taking full advantage of the situation by learning lessons from it. Passive coping strategies include (1) refusing to acknowledge the existence of stressful events, (2) giving up on making efforts to pursue the goals set under stressful situations, and (3) strengthening stressful feelings. When confronted with a single stressor or constellation of stressors, individuals are forced to consider their coping resources and select a coping response accordingly. A previous study of post-traumatic symptoms in survivors after a catastrophic earthquake associated active coping with well-being, while passive coping was often related to psychological distress [15]. Nevertheless, another study found that passive coping styles may have beneficial effects on relieving stress and temporarily coping with setbacks, suggesting that the difference between the two coping styles may be quantitative [16]. This suggests the need to explore whether these coping styles increase or reduce psychological distress during the COVID-19 pandemic.

In addition to coping strategies, effective social support has consistently been reported to protect individuals from developing mental health problems when they experience stressors [17]. Social support can be defined as a series of support measures accessible to an individual through their social relationships with other individuals, groups, and the larger community, and can be divided into three components: subjective support, objective support, and the utilization of support [18]. There are two possible mechanisms through which social support can influence mental and physical health. One is through main effects: social support is salutary for all individuals independent of the extent of stress that they are currently facing. The other mechanism is a stress-buffering model, in which the social support of others may have an ameliorating effect on life stressors, particularly for individuals under greater stress [19]. The beneficial effects of social support on psychological well-being have been widely studied and well documented across the general population and patients with various illnesses [18, 20]. The role of social support is generally beneficial and most findings demonstrate this protective role [21]. However, social support is not always beneficial, as a study found that while Asians are more likely to benefit from implicit social support (social networking), Caucasians are more likely to benefit from explicit social support (event-specific advice) [22]. The potentially complex effect of social support on psychological distress during the COVID-19 outbreak needs to be explored.

The severity of the psychological burden that COVID-19 places on the general population was not clear at the onset of the outbreak, and a model to guide successful interventions was lacking. Little is known about how Chinese are coping with the COVID-19 stressor. Based on previous studies, we aimed to investigate the psychological status of the general population in the early stages of the COVID-19 outbreak and explored factors influencing psychological distress. We further compared psychological distress, their coping style/social support and other demographic factors between the participants who did or did not suspect that they were infected. We hypothesized that an active coping style and social support were protective factors against psychological distress in the general Chinese population in the early stages of the COVID-19 epidemic. We further hypothesized that population with suspected infection would show higher psychological distress, use less active coping styles or more passive coping styles and have less social support than those without suspected infection. Our results may help provide the basis for psychological intervention programs.

## Methods

### Study participants and questionnaires

The study population comprised Chinese living in mainland China. The snowball sampling method was used to invite potential study participants. Through the WeChat application, which constitutes a mainstream medium in China, the investigators invited an initial group of 10 individuals to participate. The first set of invitees then forwarded the invitations to 10 of their contacts whom they considered suitable, and this second set forwarded the invitation in the same way. Participants filled in anonymous basic information online via the Questionnaire Star (https://www.wjx.cn), and as long as they did not report a history of serious mental illness, they were asked to provide informed consent and were able to continue to the three questionnaires (see below). The study was approved by the Ethics Committee of West China Hospital, Sichuan University. Invitees were allowed to complete the survey from 4 p.m. on February 1, 2020 until midnight on February 4, 2020.

### Instruments

First, participants filled in a custom-designed questionnaire that collected sociodemographic information about sex, age, marital status, educational level, occupation, family residence location, and family income. The questionnaire also asked about infection with SARS-CoV-2 (in the respondent or relatives), time spent searching for information about the virus everyday, history of contact with the epidemic area (Wuhan City), and presence of cases in the respondent’s community.

Then participants filled out the Mandarin versions of the six-item Kessler psychological distress scale (K6), the Simplified Coping Style Questionnaire (SCSQ), and the Social Support Rating Scale (SSRS).

The Mandarin version of the K6, which has been validated in the World Mental Health Survey [23], comprises six questions that ask respondents to rate how frequently they have felt ‘nervous’, ‘hopeless’, ‘restless or fidgety’, ‘so depressed that nothing could cheer you up’, ‘everything was an effort’, or ‘worthless’ during the past 30 days [24]. Items are rated on a five-point scale, with 0 indicating an absence of the symptom and 4 indicating that the symptom was always present during the past 30 days. The final K6 score can range from 0 to 24, with higher scores (≥13) indicating higher levels of psychological distress [25]. The K6 has shown good reliability and validity, with Cronbach’s α ranging between 0.96 and 0.97 [23].

The SCSQ [26], based on the ‘Ways of Coping’ questionnaire, is a 20-item self-report that includes dimensions of active coping (12 items) and passive coping (8 items). Responses are given on a four-point Likert scale (0 = never; 3 = very often). The instrument has been used frequently in China, with high reliability and validity [26]. The active coping dimension is composed of items 1 to 12, which mainly reflect active coping strategies an individual uses when encountering stress, such as “trying to see things in as good a way as possible” and “identifying several different ways to solve problems.” The passive coping dimension consists of items 13–20, which mainly reflect passive coping strategies that an individual uses when encountering stress, such as “relieving troubles through smoking and drinking” and “fantasizing that some miracle may happen to change the *status quo*.” The SCSQ score reflects participants’ coping style preferences, with a higher score indicating a higher possibility that the participant would adopt the relevant coping style [27]. The SCSQ has shown good reliability and validity, with Cronbach’s α ranging between 0.90 and 0.92 [26].

The SSRS is a 10-item self-report that assesses the level of an individual’s social support over the past year [28]. This measure consists of three subscales: subjective support (4 items), objective support (3 items), and utilization of support (3 items). Subjective support refers to perceived social support, meaning that people feel supported, cared for and helped by family members, friends and colleagues [e.g., Question: How many close friends do you have? Responses: (1) None, (2) 1–2, (3) 3–5, or (4) 6 or more]. Objective support refers to visible, practical and direct support (e.g., financial or other tangible resources that you received when you needed help). The utilization of support reflects the degree of social support used [Question: How do you get help when in need? Responses: (1) I am self-reliant, (2) I seldom ask for help from others, (3) I sometimes ask for help from others, or (4) I often ask for help from relatives and friends]. The total SSRS score ranges from 12 to 66 points, with higher scores indicating higher level of social support. The SSRS has shown good reliability and validity, with Cronbach’s α ranging between 0.83 and 0.86 [28].

### Quality control

Only one set of surveys was accepted from the same Internet Protocol address, and surveys were not accepted if the time to complete all questionnaires was less than 120 s. Surveys did not request any identifying information.

### Statistical analysis

All statistical analyses were performed using SPSS 21 (IBM, Armonk, NY, USA). Exploratory data analysis was conducted using frequencies for categorical variables and mean values for continuous variables. Where appropriate, odds ratios (ORs) were reported.

Differences in demographic characteristics, coping style and social support between respondents who suspected or did not suspect that they themselves had COVID-19 were assessed for significance using the independent two-samples *t* test, in the case of age and family income coefficient; or the chi-squared test, in the case of sex, marital status, education levels, residence location, presence of COVID-19 in respondent’s community, and time spent searching for information about COVID-19.

To identify predictors of high psychological distress, we classified respondents into those with high psychological distress (K6 score ≥ 13) and those with low psychological distress (K6 score ≤ 12) [24]. To identify factors influencing high psychological distress among respondents who did not suspect that they had COVID-19, we performed binary logistic regression and backward stepwise multiple logistic regression. The dependent variable was the dichotomous classification of low or high psychological distress. The model was constructed with the following covariates: age, sex, educational level, marital status, family income coefficient (total family income/number of family members), residence location (Hubei province or other), history of contact with the epidemic area (Wuhan City) or not, time spent per day searching for information about COVID-19, and questionnaire scores for positive coping style, negative coping style, subjective support, objective support and utilization of support. The least significant variables were removed one at a time until only significant variables (*P* < 0.05) remained. Supplementary Tables 1 and 2 show the factors included and excluded, respectively, in the logical regression models.

We also did group comparison to identify the risk factors associated with high psychological distress. Differences in demographic characteristics between respondents with high or low psychological distress in non-suspected cases were assessed for significance using the independent two-samples t test, in the case of age and family income coefficient; or the chi-squared test, in the case of sex, marital status, education level, residence location, presence of COVID-19 in the respondent’s community, time spent per day searching for information about COVID-19 (Supplementary Table 3).

Binary and logistic regression was not performed on data from respondents who suspected that they had COVID-19, since only one of them showed low psychological distress.

## Results

### Study population

A total of 1607 participants received the invitation to the online survey, taking a mean of 10.70 ± 7.57 min to complete all questionnaires. Three people finished in fewer than 120 s, and 16 did not finish all questionnaires. After excluding these individuals, 1588 respondents (33.12% men) were included in the final analysis. Their average age was 33.68 ± 11.96 years, 43.01% were unmarried, 8.31% had at most a senior high school level of education, 22.10% had a technical qualification, 56.68% had a bachelor’s degree, and 12.91% had a postgraduate qualification. A total of 8.80% of respondents were from Hubei Province, the initial area of the COVID-19 outbreak. Fewer than a quarter of participants (16.12%) were suspected of having COVID-19, 20.34% had a history of contact with the epidemic area, and 20.84% lived in communities where COVID-19 cases had been reported. Nearly one third (32.43%) of respondents spent more than 4 h per day searching for information about COVID-19 (Table 1). Of the 1588 respondents, 22.80% had high levels of psychological distress (K6 score ≥ 13). Mean scores were as follows: active coping style, 20.66 ± 9.42; passive coping style, 9.02 ± 4.18; subjective social support, 19.37 ± 6.72; objective support, 7.88 ± 3.92; and utilization of support, 7.07 ± 2.42 (Table 2).

**Table 1:** Demographic and clinical characteristics of the study cohort (*n* = 1588)

Characteristic	Mean ± SD	Subgroup	n (%)
Age (year)	33.68 ± 11.96	18–29	652 (41.11)
		30–39	466 (29.35)
		40–49	290 (18.26)
		50–59	130 (8.19)
		≥60	50 (3.15)
Family income coefficient	0.84 ± 0.55		
Sex		Male	526 (33.12)
		Female	1062 (66.88)
Marriage		Married	905 (56.99)
		Unmarried	683 (43.01)
Education level		Senior high school or lower	136 (8.54)
		Technical	351 (22.05)
		Bachelor	900 (56.53)
		Postgraduate	205 (12.88)
Residence in Hubei province		Yes	140 (8.80)
		No	1448 (91.20)
Suspected COVID-19		Yes	256 (16.12)
		No	1332 (83.88)
History of contact with epidemic area (Wuhan City of Hubei Province)		Yes	323 (20.34)
		No	1265 (79.66)
Living in communities with COVID-19 cases		Yes	331 (20.84)
		No	1257 (79.16)
Time spent searching for information about COVID-2019 (h/day)		1–2	766 (48.24)
		3–4	307 (19.33)
		5–6	171 (10.77)
		7–8	232 (14.61)
		> 8	112 (7.05)

Note: Family income coefficient = family income / number of people in the family

**Table 2:** Psychological distress, coping style and social support in the study cohort (*n* = 1588)

Six-item Kessler psychological distress scale	N (%)	
Score ≤ 12	1226 (77.20)	
Score ≥ 13	362 (22.80)	
**Simplified Coping Style Questionnaire**	**Mean ± SD**	**Range**
Active coping style	20.66 ± 9.42	0–36
Passive coping style	9.02 ± 4.18	0–24
**Social Support Rating Scale**	**Mean ± SD**	**Range**
Subjective support	19.37 ± 6.72	8–32
Objective support	7.88 ± 3.92	1–22
Utilization of support	7.07 ± 2.42	3–12

### Differences in demographic characteristics, coping style and social support between respondents who suspected or did not suspect that they had COVID-19

Only one of 256 respondents with suspected infection showed low psychological distress, indicating that suspected cases in our sample had high psychological distress. In contrast, only around 8% of respondents without suspected infection had high psychological distress. Respondents with or without suspected infection were different in demographic characteristics: those with suspected infection were younger (mean age, 21.20 ± 5.51), and they had lower family income (0.62 ± 0.34), higher education level, and more contact with Wuhan City. Compared to respondents without suspected infection, those with suspected infection also spent more time searching for information about COVID-19, rarely used any coping style to deal with the stressor, and had less social support (Table 3).

**Table 3:** Differences in demographic characteristics, coping style and social support between respondents who suspected or did not suspect that they had COVID-19

	Suspected (***n*** = 256)	Not suspected (***n*** = 1332)	df	***t***/***χ***^***2***^	***P***-value
Age, years	21.2 (5.51)	36.08 (11.40)	746.39	32.01	< 0.001
Family income coefficient	0.62 (0.34)	0.88 (0.57)	1090.88	70.51	< 0.001
Marriage			1	291	< 0.001
Married	234 (91.41%)	449 (33.71%)			
Unmarried	22 (8.59%)	883 (66.29%)			
Sex			1	3.13	0.08
Male	97 (37.89)	429 (32.21)			
Female	159 (62.11)	903 (67.79)			
Education level			3	45.09	< 0.001
Senior high school or lower	2 (0.78)	130 (9.76)			
Technical	63 (24.61)	288 (21.62)			
Bachelor	178 (69.53)	722 (54.16)			
Postgraduate	13 (5.08)	192 (14.41)			
Residence in Hubei province			1	320.93	< 0.001
Yes	97 (37.89)	43 (3.23)			
No	159 (62.11)	1289 (96.77)			
History of contact with epidemic area			1	1166.16	< 0.001
Yes	254 (99.22)	69 (5.18)			
No	2 (0.78)	1263 (94.82)			
Presence of COVID-19 in respondent’s community			1	100.40	< 0.001
Yes	113 (44.14)	218 (16.37)			
No	143 (55.86)	1114 (83.63)			
Time spent searching for information about COVID-2019 (h/day)			4	713.00	< 0.001
1–2	0	766 (57.51)			
3–4	22 (8.59)	285 (21.40)			
5–6	39 (15.23)	132 (9.91)			
7–8	167 (65.23)	65 (4.88)			
≥ 8	28 (10.93)	84 (6.31)			
Six-item Kessler Psychological Distress Scale					
Score ≤ 12	1 (0.39)	1225 (91.97)			
Score ≥ 13	255 (99.61)	107 (8.03)			
Simplified Coping Style Questionnaire					
Active coping style	5.50 (2.62)	23.58 (7.20)	1090.88	70.51	< 0.001
Passive coping style	5.57 (1.59)	9.69 (4.20)	1034.16	27.03	< 0.001
Social Support Rating Scale					
Subjective support	9.01 (1.49)	21.37 (5.36)	1419.38	71.06	< 0.001
Objective support	2.34 (1.13)	8.95 (3.33)	1187.55	57.46	< 0.001
Utilization of support	3.73 (0.84)	7.71 (2.07)	955.53	51.63	< 0.001

Abbreviations: df, degree of freedom

Note: Family income coefficient = family income / number of people in the family

Unless otherwise noted, values are n (%)

### Factors predicting high psychological distress in respondents without suspected infection

Binary logistic regression identified three factors that predicted high psychological distress among our respondents without suspected infection: spending > 6 h daily searching for information about COVID-19 (OR for 7–8 h, 5.26; OR for > 8 h, 7.14; both *P* < 0.001), being unmarried (OR 2.00, *P* = 0.022), and using a passive coping style (OR 1.12, *P* = 0.001). The binary logistic regression also identified four factors that predicted low psychological distress: active coping style (OR 0.88, *P* < 0.001), objective support (OR 0.89, *P* = 0.043), subjective support (OR 0.91, *P* = 0.005) and utilization of support (OR 0.86, *P* = 0.046) (Table 4). The following factors did not predict high psychological distress among non-suspected cases: age, sex, education level, family income coefficient, residence location, or history of contact with the epidemic area.

**Table 4:** Factors predicting high psychological distress in respondents who did not suspect that they had COVID-19 (n = 1332)

	95% CI		OR	β	***P***-value
	Lower	Upper			
Married					
Yes ^a^			1.0		
No	1.10	3.58	2.00	0.69	0.022
Time spent searching for information about COVID-19 (h/day)					
1-2^a^			1.0		
3–4	0.42	3.13	1.15	0.14	0.793
5–6	0. 50	2.77	1.18	0.16	0.719
7–8	2.04	12.5	5.26	1.64	0.001
≥ 8	3.23	16.7	7.14	2.00	< 0.001
Coping style					
Active	0.84	0.92	0.88	−0.13	< 0.001
Passive	1.05	1.20	1.12	0.12	0.001
Social support					
Subjective support	0.86	0.97	0.91	−0.09	0.005
Objective support	0.79	1.00	0.89	−0.12	0.043
Utilization of support	0.72	1.02	0.86	−0.15	0.046

Note: ^a^ Reference group

*Abbreviations*: *OR* odds ratio, *CI* confidence interval

In the group comparison, we did not find any significant differences in demographic features between participants without suspected infection who had high or low psychological distress, except for residence in Hubei Province (Supplementary Table 3).

## Discussion

To the best of our knowledge, this appears to be the first study to examine psychological distress in the general population in mainland China during the COVID-19 outbreak, and to investigate factors associated with that distress. The results of the present study show that in February 2020, when there were significant public concerns about the new coronavirus pandemic outbreak, 22.8% of our participants reported high levels of psychological distress (K6 score ≥ 13). Respondents with suspected infection reported higher levels of psychological distress than those without suspected infection, and the two groups differed in several sociodemographic variables, coping styles and support systems. Among those without suspected infection, factors significantly associated with high psychological distress were unmarried status, spending > 6 h per day searching for information about COVID-19, a passive coping style and lower social support.

The present study was conducted during the first two weeks of the COVID-19 outbreak, since human-to-human transmission was announced on January 20, 2020 [29]. In our study, 22.8% of participants had high psychological distress based on the cut-off score of 13 [30]. The prevalence of high psychological distress in our samples is lower than the 39.3% in a retrospective survey of perceived psychological distress in the construction industry, schools, businesses and residents in urban and rural areas during the SARS outbreak [31]. Our results may be more reliable than a retrospective study because our data were collected and analyzed during the stressor event. At the same time, the prevalence rate in our study is higher than the 17.3% of health care workers in Taiwan who showed significant mental symptoms during the SARS epidemic [32]. These findings suggest that the COVID-19 outbreak places a substantial burden on the mental health of the general population in China. Therefore, urgent measures are needed to enhance mental health services during the COVID-19 crisis.

In our study, nearly all the respondents with suspected infection showed high psychological distress, indicating high probability of having a severe mental illness and that specific intervention is needed for this subgroup [33]. This subgroup, compared to respondents without suspected infection, used less active coping styles and more passive coping styles during the outbreak; they had lower objective social support, subjective social support and utilization of social support; they were younger, had less education and lower family income; and they were more likely to be residents in Hubei Province, to have had contact with the epidemiological area (Wuhan), and to live in communities where COVID-19 cases were present. There are several explanations for the high psychological distress. It may be due to the fear of being infectious, being placed in quarantine, or suffering financial loss, stigma or discrimination. It may be due to a sense of guilt, frustration, boredom, or a feeling of having inadequate supplies or information. All these factors are stressors during the COVID-19 pandemic [34, 35]. Another potential explanation is belonging to a low-income social class, being younger, and having less education and low-skilled employment, which are risk factors for distress related to COVID-19. Indeed, being younger, being less attached to school education and belonging to a lower-income community are risk factors for distress during exposure to acute and chronic stressors in US immigrants [32, 36]. These factors may also impact social support and coping strategies: low-income, less-educated young adults are reported to lack social support and be more prone to adopting passive coping strategies, which are major risk factors for depressive symptoms in university students [37]. Finally, it is not surprising that those suspected with infection had more closely contact with Wuhan city, since COVID-19 was first observed in Wuhan. News coverage of the COVID-19 outbreak in Wuhan initially focused on the high infectivity and fatality, potentially creating fear and panic. In addition, in order to decrease the risk of disease transmission, Wuhan authorities suspended public transport indefinitely from January 23, 2020. A range of measurements were urgently adopted, such as early identification and isolation of suspected and diagnosed cases, contact tracing and monitoring, collection of clinical data and biological samples from patients, dissemination of regional and national diagnostic criteria and expert treatment consensus, establishment of isolation units and hospitals, and prompt provision of medical supplies and dispatching of external expert teams to Hubei Province [38]. The process of SARS-CoV-2 infection control and prevention involves the use of personal protective equipment, quarantine, and isolation, all of which may be further associated with fear and anxiety. It is reasonable to conclude that under these circumstances, the general population is under substantial stress and may need special care and psychological intervention.

For respondents without suspected infection, an active coping style and social support were protective factors against psychological distress in our regression model. On the other hand, unmarried status, spending more than 6 h per day searching for information about the COVID-19 outbreak, and passive coping styles were risk factors of high psychological distress.

We found that unmarried respondents were two times more likely to have increasing psychological distress in our logistic regression models. Our results are consistent with a recently published analysis in China showing that marriage was a determinant of psychological distress in the general population, and that unmarried individuals showed higher psychological distress during the COVID-19 outbreak [39]. Social support from partners and family members has been associated with well-being [40], and existing literature reveals considerable differences in social support among married, formerly married, and never-married people: unmarried people are less likely than married people to report having close family members or friends [41]. In addition, married people have larger help networks than unmarried or divorced individuals [42]. Unmarried subjects often do not benefit from the support of a spouse or family [43, 44].

Respondents without infection who showed high psychological distress (K6 score ≥ 13) also showed a higher frequency of passive coping style, such as problem-avoidance, fantasy, and self-blame. Our results are highly consistent with the findings in the subgroup of participants with suspected infection, in whom passive coping strategies were also associated with high psychological distress. Our results are also consistent with a previous meta-analysis that reported a strong association between passive coping style and depression [45]. Previous research indicated that coping styles can affect how a stressful event is perceived and how it is managed [10]. Since coping can involve “all efforts to manage taxing demands, without regard to their efficacy or inherent value” [10], it is not necessarily associated with a good outcome. Our findings are consistent with other studies that associate higher stress with greater use of emotion-oriented and social diversion-oriented coping [46].

### Coping style, social support and psychological distress

Our results emphasize the need to research coping strategies in the general population and interventions to teach coping during epidemic outbreaks. Such work may lay a solid foundation for individuals to cope positively and actively with various stress factors and circumstances [47]. Our results suggest several considerations for helping the general population handle the psychological distress caused by the COVID-19 epidemic. First, fear of COVID-19 is common in the general population worldwide, and the best way to end fear about COVID-19 is to learn about the disease and actual risk to others. Second, we should encourage people to work with colleagues to reduce financial stress: when people were unable to work during the epidemic, they may have experienced stress related to job status or financial situation. Third, providing health support, such as a telephone hotline for communication and consulting, may help reduce distress associated with social distancing, quarantine, or isolation [48]. Finally, connecting people with others for giving and receiving social support online can bolster psychological well-being. Feeling lonely and isolated from others is a common feeling during lockdown, and regularly connecting with friends and family in video or phone calls may improve the level of social support [35].

### Searching information and psychological distress

Our results indicate that media reports about how the government is fighting the outbreak, how to protect oneself from COVID-19, and how many suspected infections and cases were reported every day may engender intense confusion and panic in the general population. We suggest that the public should limit the time they spend searching for COVID-19 information to fewer than 6 h per day. Our results suggest that giving priority to traditional national media with direct connections to trustworthy medical decision-makers is associated with greater self-confidence in coping with COVID-19 [49]. Second, people can prepare daily schedules in which they ensure variety in the schedule, work, leisure, exercise, and learning. They may wish to start new activities, such as home-based “exergames” (active videogames), which may be important for maintaining physical fitness and establishing long-term adherence to exercise during the COVID-19 pandemic [50].

### Limitations

There are several limitations in our study. First, the surveying was based on network invitation rather than face-to-face random sampling, and participants had to be able to use Internet tools. Whether our results can be generalized to individuals who cannot use the Internet is unclear. Second, we did not assess whether and how respondents were engaging in prevention; preventive self-behaviors can also mediate stress levels [51]. Third, our study design was cross-sectional and so could not capture changes in psychological distress and its predictors over the course of the COVID-19 outbreak. At one year after the SARS outbreak, survivors still had elevated stress levels and disturbing levels of psychological distress [33]. Therefore, the long-term psychological implications of infectious disease outbreaks should not be ignored. Finally, 66% of our respondents were women, which may reduce the generalizability of our findings to the general Chinese population.

## Conclusions

The COVID-19 outbreak in China is substantially affecting the mental health of the general population. Mental health interventions should be implemented in a timely manner for individuals with suspected infection. Our results showed that positive coping strategies and increased social support significantly correlated with lower psychological distress. This suggests that the general population, especially those directly affected by the pandemic, should be taught active coping strategies and be encouraged to seek and maintain social support [52]. We believe that efficient mental healthcare in the national public health emergency system will empower China and the world during the campaign to contain and eradicate COVID-19 [53].

## Supplementary information

**Additional file 1 Supplementary Table 1.** Factors values assigned in the logical regression models. **Supplementary Table 2.** Factors excluded in the logical regression models. **Supplementary Table 3.** Differences in demographic characteristics between respondents with high psychological distress (HPD) and those with low psychological distress (LPD) in non-suspected cases.

## Data Availability

The identified data used in this study can be made available upon necessary request. Inquiries for the data should be sent to the corresponding authors.

## Abbreviations

COVID-19: Coronavirus disease 2019
K6: Six-item Kessler psychological distress scale
SCSQ: Simplified Coping Style Questionnaire
SSRS: Social Support Rating Scale
SARS-CoV-2: Severe acute respiratory syndrome coronavirus 2
2019-nCoV: 2019-Novel coronavirus
HIV: Human immunodeficiency virus
AIDS: Acquired immune deficiency syndrome
ORs: Odds ratios
