# Supplementary material for: Coping style, social support and psychological distress in the general Chinese population in the early stages of the COVID-19 epidemic
Source: BMC Psychiatry. 2020 Aug 27;20:426. doi: 10.1186/s12888-020-02826-3 (PMC7450895; doi:10.1186/s12888-020-02826-3)
Supplement: Supplementary file 1 — Additional file 1 Supplementary Table 1. Factors values assigned in the logical regression models. Supplementary Table 2. Factors excluded in the logical regression models. Supplementary Table 3. Differences in demographic characteristics between respondents with high psychological distress (HPD) and those with low psychological distress (LPD) in non-suspected cases. [file 12888_2020_2826_MOESM1_ESM.docx]

**Supplementary table 1** Factors values assigned in the logical regression models

|  |  |  |
| --- | --- | --- |
| **Factors** |  | **Value** |
| Sex | male | 1 |
|  | Female | 2 |
| Age group | 18-29 | 1 |
|  | 30-39 | 2 |
|  | 40-49 | 3 |
|  | 50-59 | 4 |
|  | ≥ 60 | 5 |
| Education level | Senior high school or lower | 1 |
|  | Technical | 2 |
|  | bachelor | 3 |
|  | Graduated | 4 |
| Marriage | Married | 1 |
|  | Unmarried | 2 |
| Family income coefficient |  | Primary Value |
| Residency in Hubei | Yes | 1 |
|  | No | 2 |
| Community with cases | Yes | 1 |
|  | No | 2 |
| Contact history with epidemic area | Yes | 1 |
|  | No | 2 |
| Time on CoVD-2019 | 1-2 hours per day | 1 |
|  | 3-4 hours per day | 2 |
|  | 5-6 hours per day | 3 |
|  | 7-8 hours per day | 4 |
|  | ≥8 hours per day | 5 |
| Positive coping style |  | Primary Value |
| Negative copying style |  | Primary Value |
| Subject support |  | Primary Value |
| Objective support |  | Primary Value |
| Utilization of support |  | Primary Value |

**Supplementary table 2** Factors excluded in the logical regression models

|  | β | P value | OR | 95% CI | |
| --- | --- | --- | --- | --- | --- |
|  |  |  |  | Lower | Upper |
| Being male | -0.39 | 0.20 | 0.68 | 0.38 | 1.23 |
| Age group |  |  |  |  |  |
| 18-29 |  |  | 1.0 (reference) |  |  |
| 30-39 | -0.003 | 0.99 | 0.99 | 0.23 | 4.23 |
| 40-49 | -0.28 | 0.20 | 0.68 | 0.20 | 2.83 |
| 50-59 | -0.69 | 0.13 | 0.33 | 0.13 | 2.01 |
| ≥ 60 | -1.50 | 0.02 | 0.08 | 0.04 | 1.18 |
| Education level |  |  |  |  |  |
| 1 | 0.19 | 0.64 | 1.20 | 0.55 | 2.66 |
| 2 | 0.96 | 0.17 | 2.63 | 0.67 | 10.20 |
| 3 | 0.75 | 0.34 | 2.45 | 0.45 | 9.90 |
| 4 |  |  | 1.0 (reference) |  |  |
| Family income coefficient | -0.15 | 0.60 | 0.86 | 0.49 | 1.51 |
| Residency in Hubei |  |  |  |  |  |
| No |  |  | 1.0 (reference) |  |  |
| Yes | 0.45 | 0.46 | 1.56 | 0.47 | 5.26 |
| Community with cases |  |  |  |  |  |
| No |  |  | 1.0 (reference) |  |  |
| Yes | 0.43 | 0.34 | 1.55 | 0.80 | 2.99 |
| History of contact with epidemic area |  |  |  |  |  |
| No^a^ |  |  |  |  |  |
| Yes | 0.13 | 0.83 | 1.14 | 0.34 | 3.84 |

**Supplementary table 3** Differences in demographic characteristics between respondents with high psychological distress (HPD) and those with low psychological distress (LPD) in non-suspected cases

|  | **LPD**  **(n = 1225)** | **HPD**  **(n = 107)** | **df** | ***t*/*χ^2^*** | ***P* value** |
| --- | --- | --- | --- | --- | --- |
| Age, years | 36.14 (11.39) | 35.07 (11.11) | 1330 | 0.93 | 0.35 |
| Family income coefficient | 0.82 (0.51) | 0.79 (0.49) | 1330 | 0.77 | 0.44 |
| Sex |  |  | 1 | 0.01 | 0.91 |
| Male | 394 (32.16) | 35 (32.71) |  |  |  |
| Female | 831 (67.84) | 72 (67.29) |  |  |  |
| Marriage |  |  | 1 | 0.05 | 0.91 |
| Married | 414 (33.80) | 35 (32.71) |  |  |  |
| Unmarried | 811 (66.20) | 72 (67.29) |  |  |  |
| Education level |  |  | 3 | 0.61 | 0.89 |
| Senior high school or lower | 41 (3.35) | 4 (3.73) |  |  |  |
| Technical | 80 (6.53) | 5 (4.67) |  |  |  |
| Bachelor | 928 (75.76) | 82 (76.64) |  |  |  |
| Postgraduate | 176 (14.37) | 16 (14.95) |  |  |  |
| Residence in Hubei province | |  | 1 | 29.64 | <0.001 |
| No | 1195 (97.55) | 94 (87.85) |  |  |  |
| Yes | 30 (2.45) | 13 (12.15) |  |  |  |
| Presence of COVID-19 in respondent’s community | |  | 1 | 4.16 | 0.055 |
| Yes | 193 (15.76) | 25 (23.36) |  |  |  |
| No | 1032 (84.24) | 82 (76.64) |  |  |  |

Note: Family income coefficient = family income / number of people in the family.

Unless otherwise noted, values are n (%).

Abbreviations: df, degree of freedom.
